# Heritable priming by *Trichoderma*: A sustainable approach for wheat protection against *Bipolaris sorokiniana*

**DOI:** 10.3389/fpls.2022.1050765

**Published:** 2022-12-16

**Authors:** Menka Tiwari, Rajat Singh, Rintu Jha, Prashant Singh

**Affiliations:** ^1^ Department of Botany, Institute of Science, Banaras Hindu University, Varanasi, India; ^2^ Division of Genetics, ICAR-Indian Agricultural Research Institute, New Delhi, India

**Keywords:** priming, IGIP, TGIP, Trichoderma, wheat, Bipolaris sorokiniana

## Abstract

Crop plants encounter a variety of biotic challenges in the field and faces significant reduction in crop yield. In the current scenario of an ever increasing global population, there is an urgent need to protect plant health by using sustainable approach to maximize the crop productivity and to mitigate the food demands. Nowadays, we mostly rely on chemical crop protection techniques, which are causing a number of environmental and health difficulties. Defence priming is a chemical-free, eco-friendly, and sustainable strategy of crop protection, which is also called “green vaccination. In the present study, for the first time, we used *Trichoderma* as a priming agent to protect wheat crop from spot blotch disease. We have established *Trichoderma*-mediated defence priming in wheat against *Bipolaris sorokiniana* for sustainable crop improvement. We have characterised the morphological, disease phenotype, biochemical and yield parameters of *Trichoderma*-primed and non-primed wheat under disease pressure. *Trichoderma*-primed plants were found to be more protected against *B. sorokiniana* as compared to non-primed plants. Biochemical studies indicated that there is no direct defence response after priming stimulus but the defence response was activated only after triggering stimulus in terms of enhanced defence metabolites in primed plants as compared to non-primed plants. In the present study, since defence was activated only when required, that is under disease pressure, there was no unnecessary allocation of resources towards defence. Hence, no yield penalty was shown in primed plants as compared to control. We further evaluated the inheritance of primed state to the next generation and found that progeny of primed parents also performed better than progeny of non-primed parents under disease pressure in terms of protection from *B. sorokiniana* as well as yield performance. This strategy has the potential to protect crop without any yield penalty and causing environmental degradation. Our research findings indicate that *Trichoderma*-mediated defence priming could be an alternative approach for improving wheat productivity under biotic stress. To be our best knowledge, this is the first documented report for the *Trichoderma-*mediated defence priming and induced inheritance in wheat plant. This study will open new arenas in sustainable crop protection strategies for the exploitation of defence priming in crop plants.

## Introduction

The world’s population is continually increasing, requiring us to raise the productivity of our food crops in order to properly feed the growing population (Tiwari and Singh, 2021a). Plants are continuously exposed to various pathogens, insects, pests and abiotic stresses in nature (Malook et al., 2021). Various crop protection measures have been discovered to date, such as development of transgenic crops, resistant crop varieties, and chemical insecticides and pesticides, but none of them is long-term sustainable. Each has some ethical, environmental, economic, social, or health issue (Tiwari and Singh, 2021a). In the absence of genetic resistance in crops, food production heavily depends on the use of chemicals to control pathogens. Despite their effectiveness, chemicals-based plant defence have detrimental environmental consequences and creating risks to the wider environment (Conrath et al., 2015). Modern synthetic chemicals usually have reduced environmental toxicity; however, they are expensive and only available to advanced agricultural production systems. Moreover, as with antibiotics, discovery of new chemical to control plant disease is difficult and extensive use of current agents may result in selection of pathogen strains tolerant to pesticides (Singh, 2022).

Reducing the dependence of food production on chemical control is a key goal of plant pathology research. In the present situation, researchers should emphasize the importance of understanding the natural defence system of plants in order to develop a sustainable crop protection strategy by exploiting the natural defence mechanism of the plant. There is an urgent need to develop an economical, effective and eco-friendly tool for crop improvement. Perception of certain stimuli (pathogen, pests, beneficial microbes, chemical agent, herbivory) brings the plant in a conditioned state of enhanced defensive ability against upcoming challenges, resulting in a phenotype called induced resistance (IR). It involves increased production of reactive oxygen species, enhanced callose deposition, and modulated epigenomes, transcriptomes, metabolomes and proteomes (De Kesel et al., 2021).

Plants have well developed immune systems that allow them to adapt to their surroundings and deal with stressful situations (Conrath et al., 2015). Apart from their innate immune system of controlling pre-programmed defence reactions, plants can also increase the responsiveness of their immune system in response to selected environmental signals (Hilker and Schmülling, 2019). This defensive capability of plant could be exploited by scientists to protect them from stresses. Plant defence priming is a strategy which involves pre-stressing the plant with a low dose of stress, which causes the plant to display an earlier and stronger defence response when confronted with biotic or abiotic stress (Hilker and Schmülling, 2019). Priming stimulus only brings the plant into an alert or preparatory state, but the defence is activated only upon challenge (Kerchev et al., 2020; De Kesel et al., 2021). Priming includes supporting plant defences such that they are always ready to act, but are not fully activated (yet). Plant defence priming is an intentional, regulated, and on-demand technique because in this method the plant is deliberately subjected to small dose of stress which triggers the defence response only when the plant is attacked by a pathogen (Tiwari and Singh, 2021b). The sensitization of stress responsiveness during induced resistance is called priming. Priming is one of the most economical and effective modes of resistance because it prevents wasteful metabolic consumption in plants (Mauch-Mani et al., 2017). It is a smart strategy for plant health care.

Various natural and synthetic priming agents have been used to protect the plants, such as plants growth promoting Rhizobacteria (PGPR), Jasmonic acid, Salicylic acid, Benzothiadiazol (BTH), Hexanoic acid, beta-amino butryric acid (BABA), abiotic stresses etc. (Tiwari et al., 2022). An experiment conducted on the priming of *Lycopersicon esculentum* by using *Bacillus subtilis* induced enhanced resistance upon challenge with *Alternaria solani* (Westman et al., 2019). Adss et al., 2021 reported that soyabean plants primed with rhizobacteria showed better protection from root lesion nematode *via* enhanced phytoalexin synthesis upon nematode challenge. In a study, tomato plant primed with elevated ozone exhibited enhanced resistance against phloem sucking insect *Bemisia tabaci* through increased callose deposition and activated ABA-signalling pathway (Guo et al., 2020). Similarly, priming with a mild dose of UV- B in rice provided protection against exposure to various abiotic stresses such as NaCl, PEG, and UV-B, suggesting that priming provides protection against a broad range of stresses (Thomas et al., 2020).

Plants subjected to biotic or abiotic stresses, develop long-lasting immunological memories, which enable them to exhibit stronger and faster defence responses against challenges in the future (Worrall et al., 2012). The primed state of the plant can be maintained for a lifetime even after the removal of the priming stimulus (Adss et al., 2021). This immunological memory generated in the plant due to exposure to stress and could be inherited by their progeny, which enables them to perform better under stress conditions. (Luna et al., 2012; Singh et al., 2017). One of the mechanisms responsible for the generation of this memory is epigenetic modification, which can create long-term changes in gene expression (Turgut-Kara et al., 2020). Epigenetic changes including DNA methylation, histone modifications, and RNA associated silencing are heritable to the next generation. Inheritance of epigenetic changes directly from primed generation (F0) to their offspring (F1) is called intergenerational immune priming (IGIP) and the transgenerational immune priming (TGIP) occurs when the offspring pass it down to their progeny (F2) who have not been exposed to the priming stimulus (Mauch-Mani et al., 2017; Kotkar and Giri, 2020). The inheritance of priming to the subsequent generations without any genetic alteration is known as TGIP (Mauch-Mani et al., 2017). TGIP has also been reported in various crop plants where primed plants produce resistant progeny (Walters and Paterson, 2012; Martínez-Aguilar et al., 2021). Priming of common bean with INA (2,6 dichloro-isonicotinic acid) developed long-term defence memory against *Pseudomonas syringae* pv. *phaseolicola*, which was passed down to the following generation *via* epigenetic mechanisms (Martínez-Aguilar et al., 2021). TGIP is not only restricted to herbivorous plants but also found in trees. Progeny of ink-diseased sweet chestnut trees showed boosted resistance against *Phytophthora cinnamomi* as compared to progeny of healthy trees (Camisón et al., 2019). Similarly, transgenerational induced resistance was also reported in Quercus ilex L. (holm oak). The offspring of infected Quercus exhibited increased tolerance against *Phytophthora cinnamomi* as compared to the offspring of the non-infected mother trees (Vivas et al., 2021). Defence priming rarely provides complete plant protection, but its broad-spectrum protection, long-term durability, and ability to be inherited to the subsequent generations make it appealing for integrated disease management.

Wheat (*Triticum aestivum* L.) is one of the most extensively cultivated crops throughout the world and is the second staple food of many South Asian countries including India. Wheat is cultivated due to its excellent nutritional content and wide range of commercial applications (Satti et al., 2021). It is an important source of protein and carbohydrates in developing and underdeveloped countries like India, Nepal, Bangladesh, Pakistan, etc (Singh et al., 2019). Wheat demand is expected to increase by more than 60% by 2050 due to rising population (Sahu et al., 2016). However, the wheat crop is subjected to a variety of biotic and abiotic stressors that results in significant yield losses each year. *B. sorokiniana* is one of the most devastating fungi which causes spot blotch disease in wheat crops cultivated in warm and humid regions in India and other countries (Sahu et al., 2016). The disease has caused major crop losses, particularly in India’s North Eastern Plains Zone (NEPZ), Nepal’s Tarai and Bangladesh’s north-western region (Gupta et al., 2018). Yield losses owing to spot blotch have been estimated to range from 15.5 to 19.6%, with losses reaching 100% under extreme infection situations (Kumar et al., 2020). *B. sorokiniana* is a hemibiotrophic fungus (Zhang et al., 2020). Spot blotch infection is initiated by the adhesion of conidia of *B. sorokiniana* to the leaf surface (Acharya et al., 2011). Conidia germinates on the leaf surface to form germ tube which swells within 8 hours to form appressorium (Sahu et al., 2016). Appressorium forms infecting hyphae which penetrates the cuticle of leaf within 12 hours and spread in the intercellular spaces within mesophyll tissue of leaf (Gupta et al., 2018). A new generation of conidia on conidiophores are produced within 48 hours within the leaf tissue which are capable of secondary infection. These conidia are olive-green, thick-walled, oblong, tapered towards end and have 3 to 9 septa (Gupta et al., 2018). Spot blotch symptoms most commonly appear on the leaf, sheath, node and glumes as small light brown lesions, typically oval to oblong to somewhat elliptical in shape, measuring 5–10 mm long and 3–5 mm wide (Zhang et al., 2020). These lesions are scattered throughout the leaves, have brown margins and gradually increase in size to form large necrotic patches (Gupta et al., 2018). Affected leaves lose chlorophyll and eventually die. Spikes are also affected under most severe condition of disease (Al-Sadi, 2021).

*Trichoderma* is a beneficial fungus widely used as a biocontrol agent that enhances plant growth as well as inhibits phytopathogens. (Abdullah et al., 2021). *Trichoderma*’s ability to parasitize on phytopathogenic fungus, bacteria, nematodes, and insects makes it a potential biocontrol agent (Poveda, 2021). In recent years, *Trichoderma* has also been used as a plant growth promoter which enhances the vegetative growth (root, shoot length, plant biomass, etc.) and yield of the plant (Singh et al., 2016; Pascale et al., 2017). *Trichoderma* is mainly a root endophyte which colonises the outermost layer of the plant root and activates systemic plant defences against the attack of pests and/or pathogens (Poveda et al., 2020).

In past research, *Trichoderma* has been used as a biocontrol agent for crop improvement, but less is known about the potential of *Trichoderma* to prime resistance to biotic stresses in wheat, such as attack by pathogens. We have used *Trichoderma* for the first time as a defence priming agent in wheat against biotic stress. The present study was carried out to check the establishment of *Trichoderma*-mediated defence priming in wheat against spot blotch disease as well as the inheritance of primed state to the next generation by analysing the morphological, disease phenotype, biochemical and yield parameters. The opportunity to increase resistance to pests and diseases through priming creates a new process by which dependency on chemicals can be reduced without altering the genetic make-up of our elite crop varieties. The current study might be a non-pesticide alternative for sustainable crop protection strategy.

## Materials and methods

### Materials

Wheat (HUW-510) seeds were obtained from the Department of Genetics and Plant Breeding, Institute of Agricultural Sciences, B.H.U. Varanasi. It is a late-sown variety, released in 1999/2000, and susceptible to spot blotch disease caused by *B. sorokiniana*. The priming agent, *Trichoderma asperellum* (T42; GenBank accession JN 128894), was obtained from Hoffmann Laboratory, Department of Mycology and Plant Pathology, Institute of Agricultural Sciences, B.H.U., Varanasi. The pathogen, *B. sorokiniana* HD3069 (accession no.-MCC 1572), was obtained from the Department of Mycology and Plant Pathology, Institute of Agricultural Sciences, BHU.

### Methods

#### Culturing of microorganisms

The potato dextrose agar (PDA) medium was prepared and autoclaved at 121.6°C, 15 psi pressure, for 20 minutes. PDA media (20ml) was poured into each sterilised petri plate and allowed to solidify. A 5mm mycelial culture bit was cut with the help of a cork borer from a 7-days old culture of *Trichoderma asperellum* T42. A culture bit was placed in the middle of petri plates filled with media. Plates were incubated at 27 ± 2° C in a BOD incubator for 7 days until sporulation.

Likewise, *B. sorokiniana* HD 3069 was also inoculated and incubated at 25 ± 2°C in a BOD incubator for 15 days until sporulation.

#### Preparation *of Trichoderma* spore suspension and *B. sorokiniana* inoculum

Spore suspension of *Trichoderma* was prepared from 7 days old culture. Sporulated Petri plate of *Trichoderma* was filled with sterilised distilled water and the spores were scraped using an autoclaved glass spreader. The spore suspension was filtered using two-layered sterile muslin cloth, and further, the filtered suspension was diluted with sterilised distilled water to adjust the final concentration to 1 × 10^7^ spore/ml by counting with a haemocytometer.

Conidia of *B. sorokiniana* HD 3069 were harvested from a 15-day old culture plate by filling the petri plate with 10 ml of sterilised distilled water and scraping the conidia gently with an autoclaved glass spreader. The resulting conidial suspension was filtered by using two-layered sterile muslin cloth and the conidial suspension was adjusted to a concentration of 3 x 10 ^4^ conidia/ml.

#### Seed priming and plant growth

The wheat seeds were surface sterilised by using 1% sodium hypochlorite solution and washed three times using sterilised distilled water. Surface sterilised seeds were immersed in prepared *Trichoderma* spore suspension (1 × 10^7^ spore/ml) for 12 hours. Seeds immersed in distilled water were taken as a control. After 12 hours, both *Trichoderma*-primed and non-primed seeds were placed on sterilised moist filter paper in a petri plate for germination. After 5 days, the seeds were sown in pots filled with sterilised garden soil mixed with vermicompost (3:1) to grow G0 generation. Pots were kept in a growth chamber with a 25/20°C day/night temperature and a 16-hour light to 8-hour darkness photoperiod, 70% relative humidity, and a photon flux density of 300 mol m^-2^ s^-1^. The experiment was repeated three times independently and each set of treatments contained three pots with seven plants in each pot (n=21).

#### Measurement of morphological parameters

Plants were carefully dug and uprooted after 30 days of sowing and washed thoroughly with water in order to remove soil attached to the roots. Growth parameters such as root length, shoot length, leaf area, root fresh wt., shoot fresh wt., root dry wt., shoot dry wt. were quantified. Total leaf area was measured by using a leaf area metre. Root and shoot biomass were determined by separating root and shoot portions and oven drying them at 50°C until the weight became consistent.

#### Challenging wheat plant with conidial suspension of *B. sorokiniana* after 30 days of sowing

Wheat was primed with *Trichoderma* spore suspension (1 × 10^7^ spore/ml) at seed stage and sown in pots. Primed as well as non-primed wheat plants were challenged with *B. sorokiniana* HD 3069 after 30 days of sowing by foliar spray of conidial suspension (3 ×10 ^4^ conidia/ml) of *B. sorokiniana*. Tween 20 (0.1%) was added as a surfactant before spraying on the wheat plants. Inoculated plants were covered with transparent plastic to maintain the humidity. Symptoms were observed on the leaves after 7 days of inoculation. Wheat leaves with symptoms were observed under a compound microscope.

The treatments were control (C), only *Trichoderma-*primed (T), *Trichoderma-*primed and challenged with *B. sorokiniana* (T+B), non-primed and challenged with *B. sorokiniana* or only *B. sorokiniana* (B).

#### Disease assessment

The severity of the disease was determined using a standard area diagram (SAD) prepared from wheat leaves with varying levels of symptoms.

No symptom – Level 0, 1 to 20% - Level 1, 20 to 40% - Level 2, 40% to 60% - Level 3, 60% to 80% - Level 4, 80% to 100% - Level 5

Percent disease index (PDI) was calculated by using the following formulae (Sultana et al., 2018)


$$PDI=\frac{\text{Sum of all disease rating}\times 100}{\text{Total number of ratings}\times \text{Maximum grade}}$$

### Biochemical parameters

#### Chlorophyll and carotenoids

The amounts of chlorophyll and carotenoid were estimated by using formulae given by Maclachlan and Zalik (1963) and Duxbury and Yentsch (1956), respectively. A leaf sample of 0.1 g was taken and crushed in 10 ml of 80% acetone. Then centrifugation was done at 6000 rpm for 15 minutes and the OD of the supernatant was taken at 480 nm, 510 nm, 645 nm, and 663 nm using a UV-2600 UV–VIS Spectrophotometer, Shimadzu (Japan). The amounts of chlorophyll a, chlorophyll b, and carotenoids were estimated by using the following formulae.


$$\text{Amount of chl a }\left(\text{mg/g}\right)=\frac{(12.3\times OD_{663}-\;0.86\times OD_{645})\;V}{1000\times \text{w}\times \text{d}}$$

$$\text{Amount of chl b }\left(\text{mg/g}\right)=\frac{\left(19.6\times {\text{OD}}_{645}–\;3.6\times {\text{OD}}_{663}\right)}{1000\;\times \text{w }\times \text{d}}$$

$$\text{Carotenoids }\left(\text{mg/g}\right)=\frac{(7.6\times {\text{OD}}_{480}–\;1.49\times {\text{OD}}_{510})\text{ V}}{1000\times \text{w}\times \text{d}}$$

Where, d = path length of light

w = weight of leaf sample

V = volume of extract

#### Malondialdehyde content

MDA content was determined by the method given by Heath and Packer (1968). 0.1 g of leaf was homogenised in 5 ml of 5% TCA (Trichloroascetic acid) and centrifuged at 10,000 rpm for 20 minutes. 1 ml of supernatant was taken and mixed with 4 ml of 0.5% TBA (Thiobarbituric acid) in a 20% TCA solution. The solution was heated in a water bath for 30 minutes and cooled on ice immediately, and OD was taken at 532 nm and 600 nm.


$$\text{MDA content }\left(\text{n mole/mg fresh wt}.\right)=\frac{({\text{A}}_{532}–{\text{A}}_{600})\times 10^{3}}{155}$$

A_532_ is absorbance at 532 nm of wavelength

A_600_ is absorbance at 600 nm of wavelength

#### Total ROS scavenging activity

Total ROS (reactive oxygen species) scavenging activity was determined by the method given by Blois (1958). 0.1 g of leaf samples were homogenised with 10 ml of 90% methanol and centrifuged at 6000 rpm for 15 minutes. 1 ml of extract was mixed with 1 ml of 0.3 mM DPPH (2,2 diphenyl-1-picryl hydrazyl) and 1 ml of methanol (90%) and kept in the dark for 30 minutes. Then the OD was taken at 515 nm.


$$\text{Total ROS scavenging activity}=\frac{{\text{A}}_{o}-{\text{A}}_{1}}{{\text{A}}_{o}}\times 100$$

A_o_ = Absorbance of control at 515 nm of wavelength.

A_1_ = Absorbance of sample at 515 nm of wavelength.

#### Total phenol

Phenol was estimated by the method given by Bray and Thorpe (1954). 0.1 g of leaf sample was crushed with 10 ml of acetone (80%) and centrifuged for 15 minutes at 6000 rpm. 1 ml of supernatant was mixed with 1 ml of Folin-Ciocalteaus reagent (1N) and 2 ml of Na_2_CO_3_ (5%). Then the final volume was made up to 10 ml with distilled water. Then the mixture was heated in a water bath till a blue colour appeared, which was then cooled to room temperature. OD was taken at 650 nm using spectrophotometer.


$$\text{Phenol content }\left(\text{mg/g fresh leaf}\right)=\frac{µg\text{ phenol}\times V}{\text{w}\times \text{v }\times 1000}$$

Where V = vol. of extract

w = wt. of leaf sample

v = vol. of supernatant

#### Ascorbic acid

Ascorbic acid content was determined by the method given by Keller and Schwager (1977). 0.1 g of leaf was homogenised in 10 ml of extraction solution (0.05% oxalic acid and 0.075% EDTA) and centrifuged at 6000 rpm for 15 minutes. 1 ml of supernatant was mixed with 5 ml of DCPIP (20 g/ml) (Dichlorophenolindophenol). Then a pink colour appeared, and OD was taken at 520 nm (Es). Then 1 drop of 1% ascorbic acid was added and the OD of the bleached solution was taken at 520 nm (Et). OD of the blank solution was taken at 520nm (Eo).


$$\text{Ascorbic acid content}=\frac{\text{Eo}–\text{Es}–\text{Et}\times \text{V}}{\text{w}\times 100\times \text{v}}$$

V = Total volume of extract made.

w = weight of leaf sample

v = volume of extract taken

Es = OD of pink coloured sample

Et = OD of sample bleached by 1% ascorbic acid

Eo = OD of blank solution

#### Yield-related traits

At maturity, the wheat spikes were harvested and the following parameters were recorded:

Spike length was measured using a centimeter scale. Grain width and grain length were measured by using Image J software. Weight of each 1000 grain of wheat was measured by using analytical balance. Total no. of spikes was measured by counting the total no. of spikes in each treatment.


$$\text{Spikelet density}=\frac{\text{spikelet number per spike}}{\text{spike length}}$$

$$\text{Spikelet fertility}\left(\text{in}\%\right)=\frac{\text{fertile spikelet number per spike}\times 100}{\text{total spikelet number per plant}}$$

#### Intergenerational immune priming

Seeds of different treatments (C, T, B, and T+B) of the parent (G0) generation were collected for evaluation of IGIP. Collected seeds were surface sterilised by using 1% sodium hypochlorite solution and washed three times with sterilised distilled water. Seeds were soaked in distilled water for 12 hours before being placed in petri plates on sterile moist filter paper for germination. Germinated seeds were sown in pots containing sterilised garden soil mixed with vermicompost (3:1) to grow G1 generation of wheat. Pots were kept in a growth chamber with a 25/20°C day/night temperature and a 16-hour light to 8-hour darkness photoperiod, 70% relative humidity, and a photon flux density of 300 mol m^-2^ s^-1^. The progeny (G1) plants were challenged with *B. sorokiniana* HD 3069 by foliar spray of spore suspension (3 x 10 ^4^ conidia/ml) after 30 days of sowing. Pots were covered with transparent plastic to maintain humidity. Symptoms were observed after 7 days of inoculation and percent disease index was calculated. Biochemical as well as yield parameters were also estimated in G1 wheat after *B. sorokiniana* challenge. Outline of the experimental design of the present study is given in **Supplementary Figure 3**.

### Statistical analysis

All the experiments were carried out in three independent biological replicate. Scientific data analysis software SPSS 21.0 (IBM Corp, New York) was used to apply one-way analysis of variance (ANOVA) to identify the significant effect of *Trichoderma* priming on the different biochemical and yield parameters of wheat under disease pressure. The levels of significant differences between different treatments were determined using Tukey’s *post hoc* test at p< 0.05 probability threshold. Additionally, the significantly different mean for different parameters (seed germination percentage, growth parameters and percent disease index) between *Trichoderma*-primed and non-primed wheat were tested using the Student’s t-test.

## Results

### Effect of *Trichoderma* priming on seed germination

**Figure 1:**
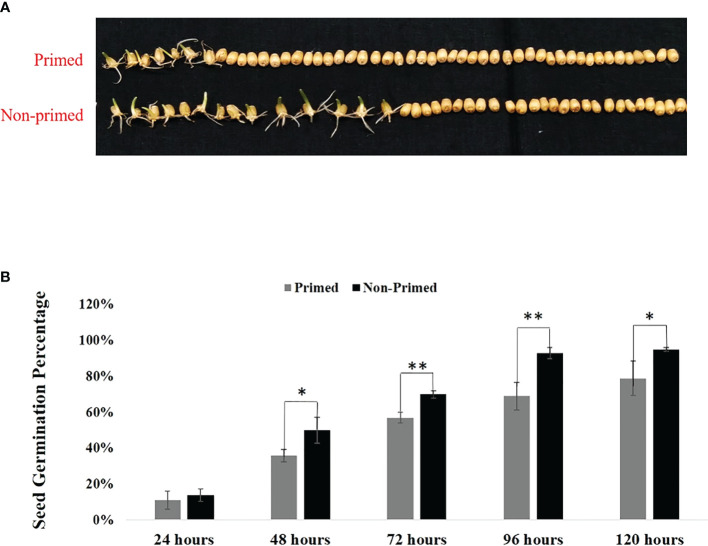
Germination of *Trichoderma* primed and non-primed wheat seeds in parent (G0) generation. **(A)** Germination of *Trichoderma* primed and non-primed wheat seeds after 48 hrs. **(B)** Seed germination percentage of primed and non-primed wheat seeds after 120 hrs. (11—50). Data in **(B)** is represented as mean ± SD, and P values (*P< 0.05 and **P< 0.01) are indicated by student's-t test. Results are representatives of three independent biological replicates. n=50.

Seed germination was evaluated in *Trichoderma* primed and non-primed wheat seeds at different time intervals. The results shown in **Figures 1A, B** indicate that seed germination was reduced by 16% in primed wheat seeds compared to non-primed wheat seeds after 120 hours, indicating that at the initial stage, there is a little bit of cost associated with *Trichoderma* priming.Effect of *Trichoderma* priming on the above and below ground growth parameters of wheat

**Figure 2:**
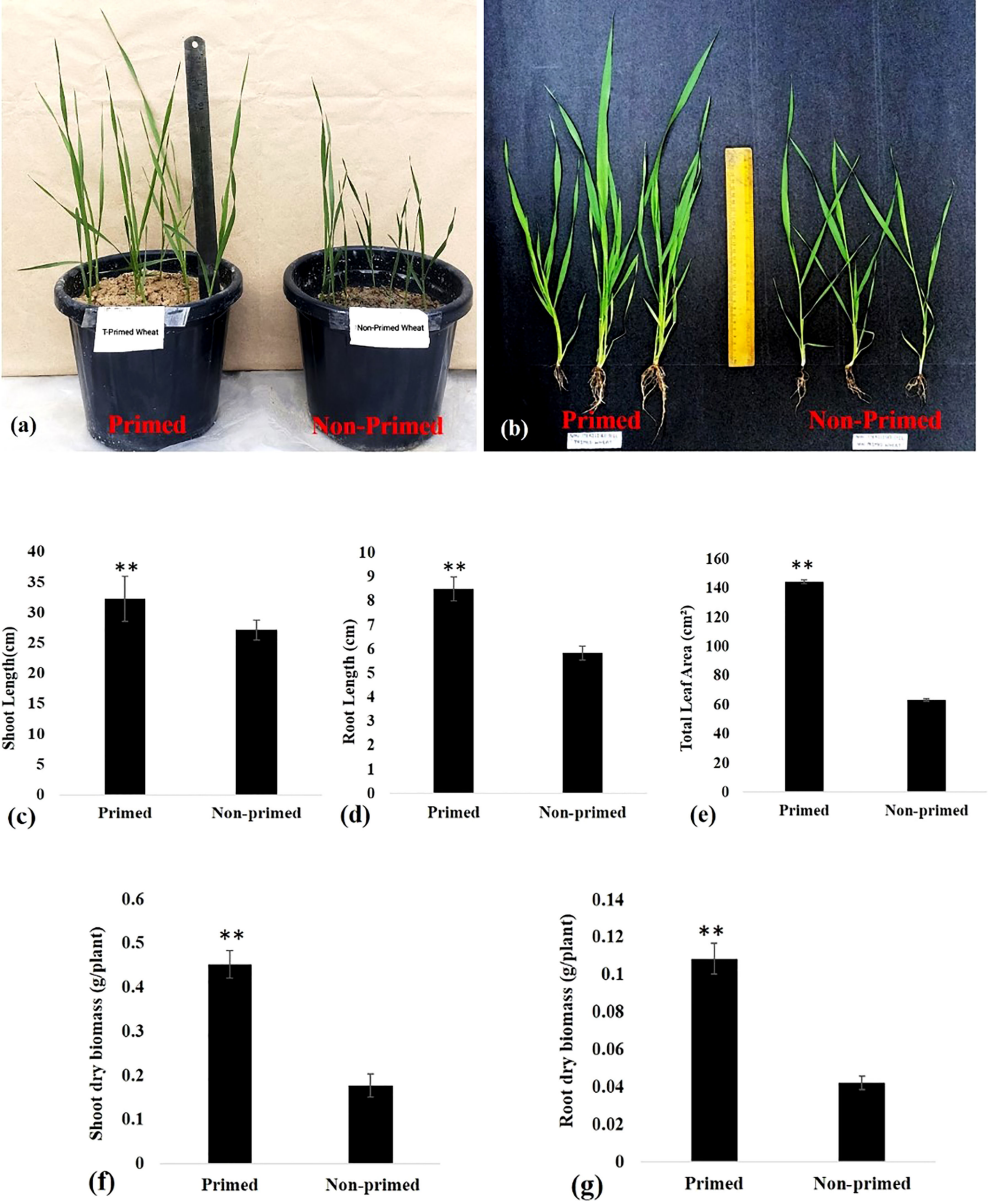
Growth of *Trichoderma* primed and non-primed wheat plants in G0 generation after 30 days of sowing **(A)** Plant height of *Trichoderma* primed and non-primed wheat plant. **(B–D)** Shoot length and root length of *Trichoderma* primed and non-primed wheat plant. **(E)** Total leaf area, **(F)** Shoot dry biomass, **(G)** Root dry biomass. Data in c, d, e, fand g are represented as mean ± SD, and P values (**P< 0.01) are indicated by student's t-test. Results are representatives of three independent biological replicates. n=21.

There was a significant increase in shoot and root length by 18.91% and 45.79%, respectively in primed plants compared to non-primed plants after sowing germinated seeds in the soil (**Figures 2A–D**). Total leaf area of primed plants was found 2.28 times more than non-primed wheat plants (**Figure 2E**). Shoot dry weight and root dry weight were enhanced by 2.56 and 2.58 times, respectively, in primed wheat as compared to non-primed wheat (**Figures 2F, G**).

### Disease assessment of primed and non-primed wheat plant

**Figure 3:**
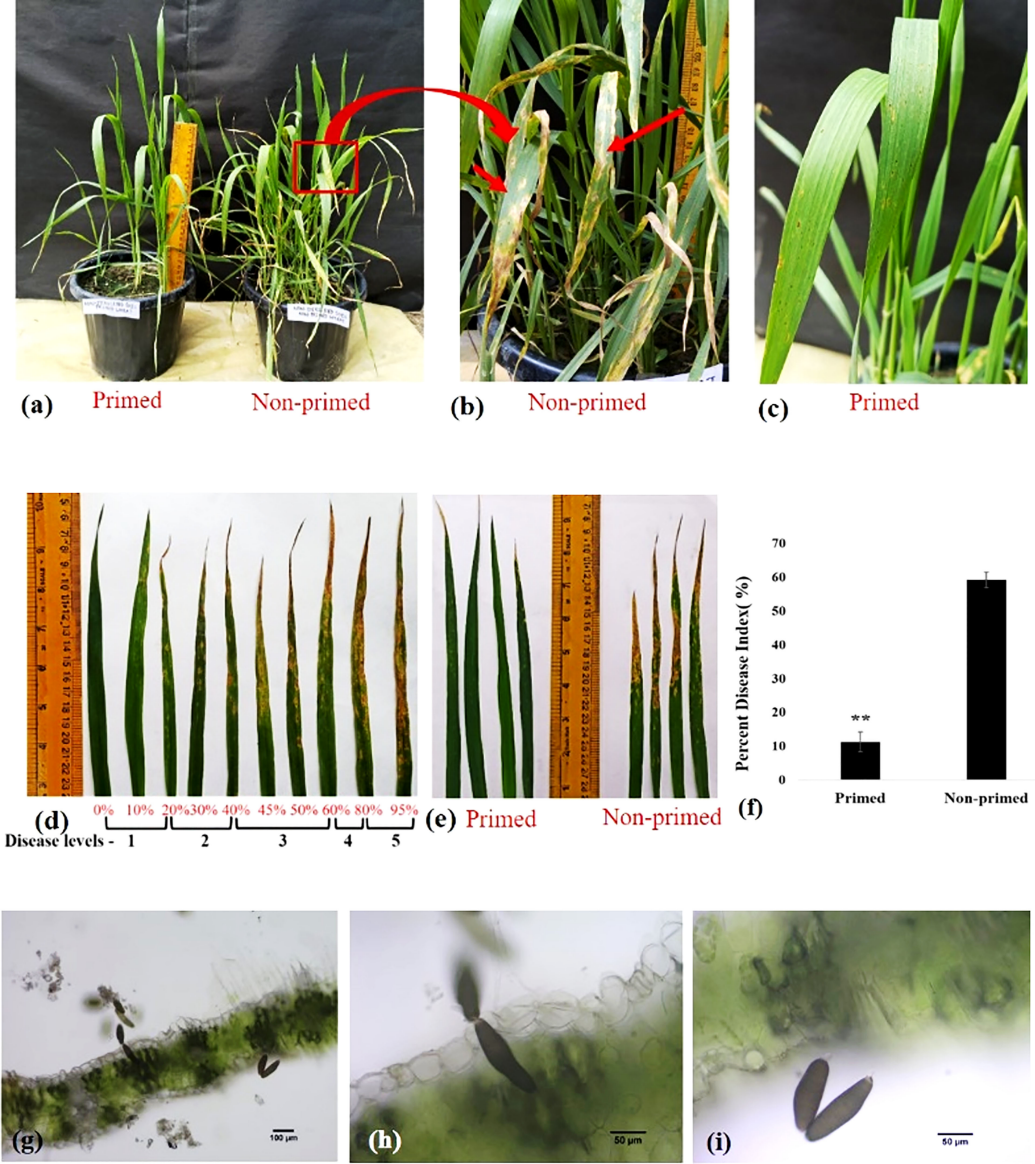
Disease assessment of spot blotch disease in wheat in G0 generation. **(A–C, E)** Symptoms of spot blotch disease on primed and non-primed wheat leaf after challenging with *Bipolaris sorokiniana* HD 3069. In figure b red arrow indicate symptoms of spot blotch. **(D)** Standard area diagram (SAD) of spot blotch disease in wheat after challenging with *Bipolaris*. **(F)** Percent disease index of primed and non-primed wheat after challenging with *Bipolaris*. **(G–I)** Conidia of *Bipolaris* on the wheat leaves 7 dpi under microscope in 10x **(G)** and 40x **(H, I)**. Results are representatives of three independent biological replicates. **P< 0.01.

*Trichoderma* priming significantly lowered the severity level of spot blotch disease. Disease symptoms were reduced in primed wheat as compared to non-primed wheat after challenging with *B. sorokiniana* HD 3069 (**Figures 3A–E**). Percent disease index was measured 7 dpi (days post inoculation) by using SAD (**Figure 3D**). Quantification of percent disease index clearly indicated that *Trichoderma*-primed wheat plants were 5.23 times more protected as compared to non-primed wheat plants against *B. sorokiniana* (**Figure 3F**). Microscopic analysis showed conidia of *B. sorokiniana* on wheat leaves under different magnification (**Figures 3G-I**). Results of disease assessment suggest that *Trichoderma* priming has enabled wheat plants to combat the *B. sorokiniana* challenge.

### Biochemical changes in primed and non-primed wheat after challenging with *B. sorokiniana*

**Figure 4:**
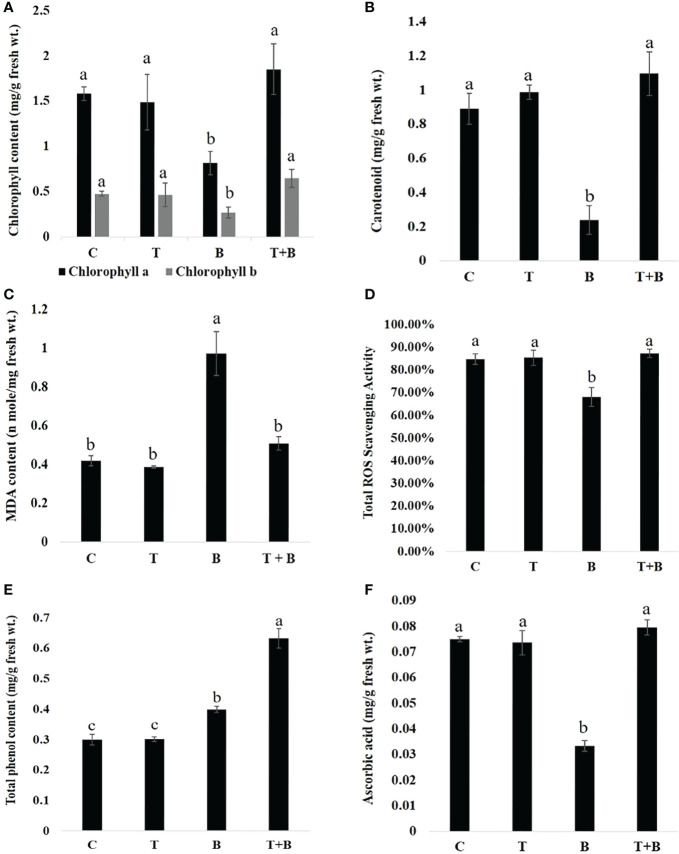
Biochemical changes in primed and non-primed wheat (G0) after challenging with *Bipolaris sorokiniana* HD 3069. **(A)** chlorophyll a and chlorophyll b, **(B)** carotenoid , **(C)** MDA content, **(D)** ROS scavenging activity, **(E)** Total phenol content and **(F)** Ascorbic acid of primed and non-primed wheat after challenging with *Bipolaris*. C = control, T = *Trichoderma-primed*, B = Non-primed wheat challenged with *Bipolaris*, T+B *Trichoderma*-primed and challenged with *Bipolaris*. Data in **(A–F)** are represented as mean ± SD (n = 21), and different alphabets indicate statistically significant difference at P<0.05 in Tukey's test. Results are representatives of three independent biological replicates.

Biochemical parameters were quantified in primed (T, T+B) and non-primed (C, B) wheat after challenging with *B. sorokiniana* and significant differences were found as shown in **Figure 4**. Quantification of photosynthetic pigments showed that chlorophyll a and b (**Figure 4A**) content was enhanced significantly by 2.28 and 2.39 times in primed plants (T+B) as compared to non-primed (B) after challenging with *B. sorokiniana.* However, no significant difference was observed between control (C) and only primed (T) wheat plants, indicating that defence is not activated after priming stimulus but activated only after triggering stimulus. In addition to that, carotenoid content (**Figure 4B**) was also quantified and found to be 4.56 times more in T+B as compared to B, but the level of carotenoid was found to be almost similar in treatments C and T.

Wheat plants primed with *Trichoderma* and challenged with *B. sorokiniana* (T+B) showed 1.9 times less MDA content (**Figure 4C**) than non-primed (B) plants challenged with *B. sorokiniana.* MDA content in T+B was found to be almost similar to non-challenged (C, T) plants. It shows that primed plants (T+B) after challenge are almost as protected as non-challenged plants (C, T). We also measured ROS scavenging activity (**Figure 4D**) in primed and non-primed wheat after *B. sorokiniana* challenge and found a nearly identical trend. The T+B treatment showed 1.2 times enhanced ROS scavenging activity as compared to treatment B, but the level of ROS scavenging activity was found to be similar in C, T, and T+B plants. Total phenol content (**Figure 4E**) was found to be enhanced by 1.57 times in T+B treatment as compared to B, but the level of phenol was found to be similar in C and T. The level of ascorbic acid was found to be augmented by 2.6 times in the T+B treatment as compared to B. However, the same was found to be similar in C, T, and T+B plants (**Figure 4F**). All these results clearly indicate that *Trichoderma*-mediated priming is one of the most economical and effective modes of resistance because it prevents wasteful metabolic consumption in plants.

### Impact of *Trichoderma* priming on yield-related traits of wheat

**Figure 5:**
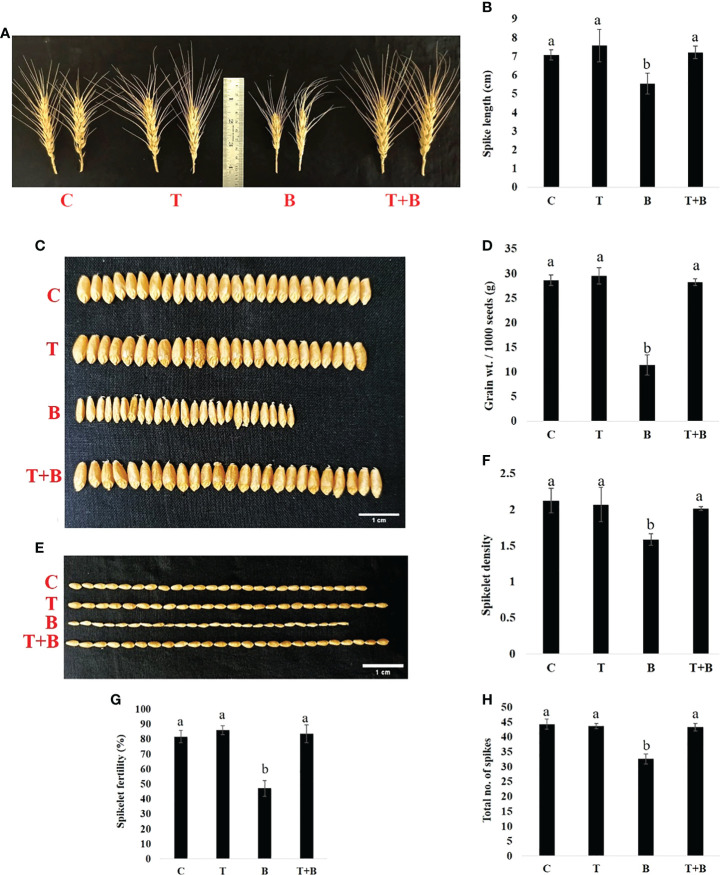
Effect of *Trichoderma*-priming on the yield of the wheat in G0 after challenging with *Bipolaris sorokiniana* HD 3069. **(A, B)** Spike length, n =21 **(C)** Grain width. Scale bar = 1 cm, n = 25, **(D)** Grain weight / 1000 seeds (n=1000) **(E)** Grain length. Scale bar =1 cm, n = 25, **(F)** Spikelet density; **(G)** Spikelet fertility; and **(H)** Total no. of spikes of wheat after challenging with *Bipolaris*. C = control, T = *Trichoderma*-primed, B = Non-primed wheat challenged with *Bipolaris*, T+B = *Trichoderma*-primed and challenged with *Bipolaris*. Data in **(B, D, F–H)** are represented as mean ± SD and different alphabets indicate statistically significant difference at P<0.05 in Tukey's test. Results are representatives of three independent biological replicates. (n = 21).

Furthermore, at maturity, different yield parameters of the primed and non-primed wheat were measured. As shown in **Figure 5**, *Trichoderma*-primed wheat produced more yield as compared to non-primed wheat under disease pressure. The spike length of wheat was found to be enhanced significantly by 30.14% in T+B wheat as compared to B, and the spike length of T+B wheat was found to be nearly identical to that of non-challenged wheat (C and T) (**Figures 5A, B**). Grain width and grain length of wheat were also found to be enhanced significantly by 52% and 19.64%, respectively, in T+B treatment as compared to B (**Figures 5C, E** and **Supplementary Figure 1**). Grain weight was found to be 2.25 times more in T+B as compared to B (**Figure 5D**). Spikelet density and fertility were found to be 1.27 and 1.76 times higher respectively in T+B than in B. (**Figures 5F, G**). The total no. of spikes in T+B was significantly enhanced by 1.58 times as compared to treatment B (**Figure 5H**). All these results clearly suggest that *Trichoderma* priming enhances wheat productivity under disease pressure.

### Intergenerational immune priming in wheat

**Figure 6:**
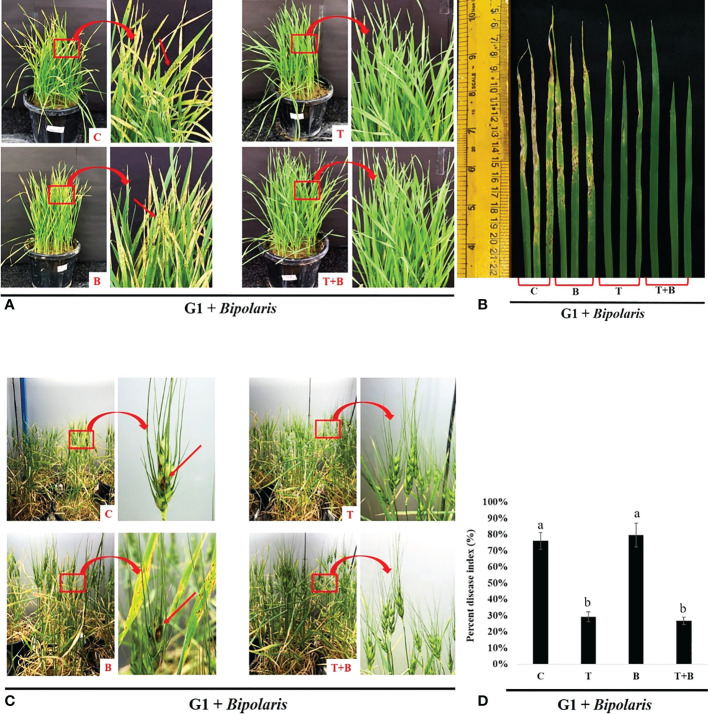
Disease assessment of spot blotch disease in the progeny (G1) of primed and non primed wheat (G0). **(A, B)** Symptoms of spot blotch disease on the leaves of G1 of primed and non-primed wheat (G0) after challenging with *Bipolaris sorokiniana* HD 3069. **(C)** Symptoms of spot blotch disease on spike of Gl of primed and non-primed wheat (G0) after challenging with *Bipolaris*. **(D)** Percent disease index of G1 wheat after challenging with *Bipolaris*. (G1 + *Bipolaris*) = progeny of G0 challenged with *Bipolaris sorokiniana* HD 3069. In figure a and c red arrow indicate symptoms of spot blotch. C = control, T = *Trichoderma*-primed, B = Non-primed wheat challenged with *Bipolaris*, T+B = *Trichoderma*-primed and challenged with *Bipolaris*. Data in d is represented as mean ± SD and different alphabets indicate statistically significant difference at P<0.05 in Tukey's test. Results are representatives of three independent biological replicates. n = 21.

**Figure 7:**
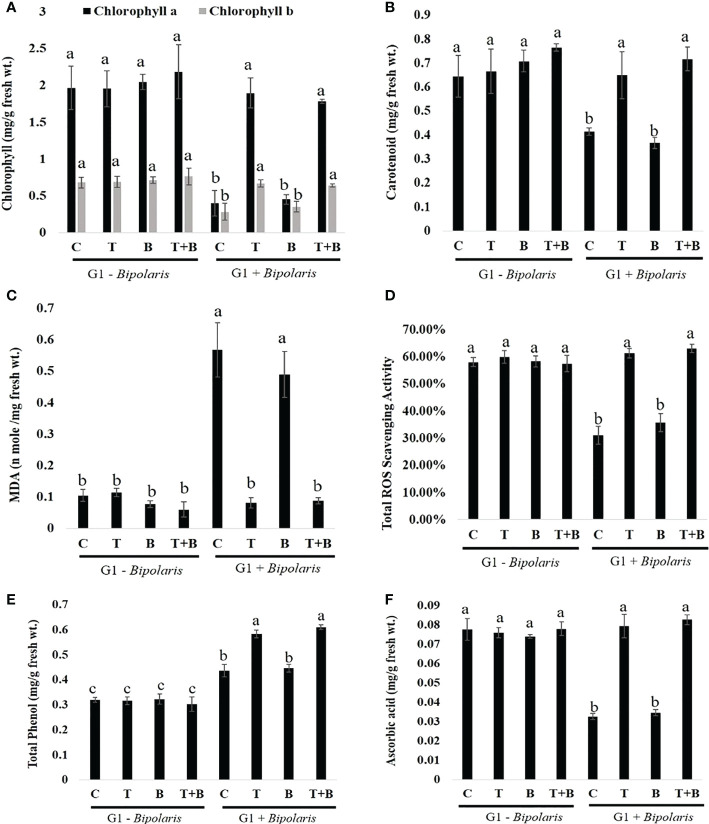
Effect of intergenerational immune priming on biochemical changes in progeny (G1) of primed and non-primed wheat (G0) after challenging with *Bipolaris sorokiniana* HD 3069. **(A)** chlorophyll a and chlorophyll b, **(B)** carotenoids, **(C)** MDA **(D)** ROS scavenging activity **(E)** Total phenol and **(F)** Ascorbic acid of G1 after challenging with *Bipolaris sorokiniana* HD 3069. C = control, T Trichoderma-primed, B = Non-primed wheat challenged with *Bipolaris*, T+B = *Trichoderma*-primed and challenged with *Bipolaris*. (G1 - *Bipolaris*) = progeny of G0 not challenged with Bipolaris sorokiniana HD 3069, (G1 + *Bipolaris*) = progeny of G0 challenged with *Bipolaris sorokiniana* HD 3069. Data in **(A–F)** are represented as mean ± SD (n=21), and different alphabets indicate statistically significant difference at P<0.05 in Tukey's test. Results are representatives of three independent biological replicates.

In our study, we also investigated the inheritance of primed state to the subsequent generation. We collected the seeds from the parent (G0) generation and sowed them in the pots containing sterilised soil to raise progeny (G1) generation. G1 plants were challenged with *B. sorokiniana* after 30 days of sowing. Progeny of primed wheat showed more protection against the pathogen, *B. sorokiniana*, as compared to the progeny of non-primed wheat (**Figures 6**, **7**). Progeny of only primed wheat (T) as well as progeny of primed wheat challenged with *B. sorokiniana* (T+B) showed fewer symptoms on their leaves as compared to the progeny of control wheat (C) and only *B. sorokiniana* challenged wheat (B) (**Figures 6A, B**).

Progeny of primed wheat (T and T+B) also showed fewer symptoms on the spike during grain filling as compared to progeny of non-primed wheat (C and B) (**Figure 6C**). Percent disease index was also reduced in progeny of T and T+B wheat as compared to progeny of C and B wheat, suggesting that primed state is inherited to the next generation of wheat (**Figure 6D**).

Biochemical parameters were evaluated in G1 plants after 7 days of challenging with *B. sorokiniana* HD 3069 as shown in **Figure 7**. Chlorophyll a and b content was significantly enhanced in progeny of primed wheat (T and T+B) as compared to progeny of non-primed wheat (C and B) (**Figure 7A**). However, no significant difference was observed in chlorophyll level between progeny of T and T+B indicating that progeny of only primed wheat (T) as well as progeny of primed and challenged wheat (T+B) are showing same level of protection from *B. sorokiniana*. Similarly, carotenoid content was increased in the progeny of T and T+B when compared to the progeny of C and B after *B. sorokiniana* challenge (**Figure 7B**). In addition to that, MDA content was reduced significantly in progeny of T and T+B as compared to progeny of C and B (**Figure 7C**). However, the level of MDA was found to be almost similar in progeny of treatment T and T+B as well as in the progeny of treatment C and B. We then measured the levels of ROS scavenging activity, total phenol, and ascorbic acid and discovered that the content of all three parameters was significantly higher in the progeny of T and T+B compared to the progeny of C and B, implying that defence priming is passed down to the next generation (**Figures 7D–F**).

**Figure 8:**
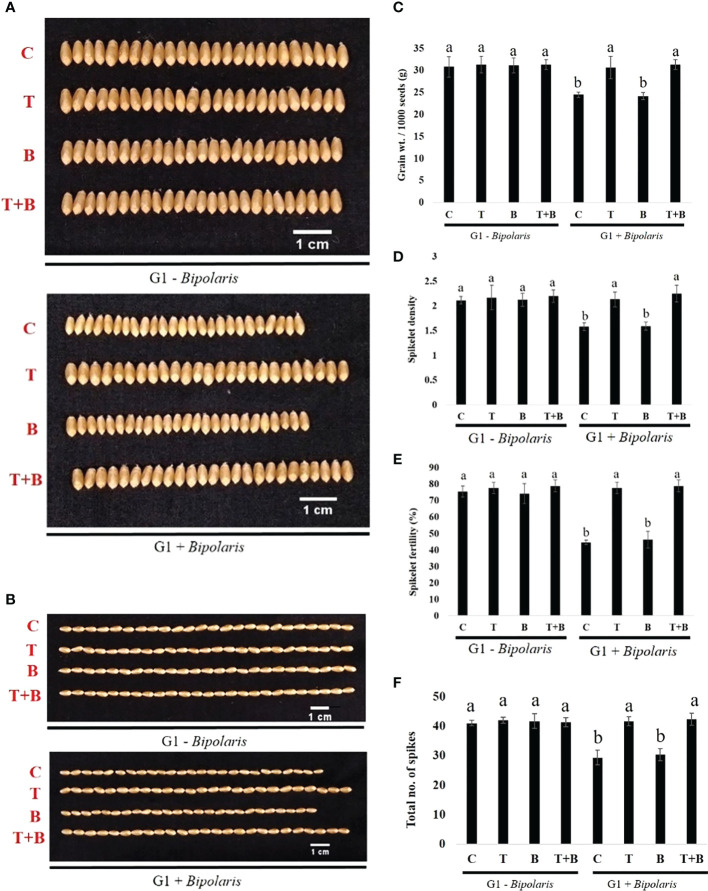
Effect of intergenerational immune priming on the yield of the G1 wheat after challenging with *Bipolaris sorokiniana* HD 3069. **(A)** Grain width. Scale bar = 1 cm, n = 25, **(B)** Grain length. Scale bar = 1 cm, n = 25, **(C)** Gram weight / 1000 seeds (n = 1000) **(D)** Spikelet density, **(E)** Spikelet fertility and **(F)** Total no. of spikes of wheat after challenging with *Bipolaris*. C = control, T *Trichoderma*-primed, B = Nonprimed wheat challenged with *Bipolaris*, T+B = *Trichoderma*-primed and challenged with *Bipolaris*. Data in **(C–F)** are represented as mean ± SD and different alphabets indicate statistically significant difference at P<0.05 in Tukey's test. Results are representatives of three independent biological replicates. (n = 21).

Measurement of yield-related traits (total no. of spikes, spike length, spikelet density, spikelet fertility, grain width, grain length, grain weight after maturity) of progeny of primed (T, T+B) and non-primed (C, B) wheat was carried out as shown in **Figure 8** and **Supplementary Figure 2**. We discovered that the productivity of progeny of primed (T, T+B) wheat was higher than that of non-primed (C, B) wheat in all parameters after challenge with *B. sorokiniana.* The above results assure us that *Trichoderma* priming was inherited to the next generation of wheat, which enabled the progeny of primed wheat to perform better than the progeny of non-primed wheat under biotic stress.

## Discussion

Plants are subjected to a variety of biotic stresses in their natural settings (Westman et al., 2019). Defence priming is a sustainable strategy to protect crops against stresses. Pathogens, beneficial microorganisms, abiotic stresses, and chemicals could all be used to prime plants for resistance (Mauch-Mani et al., 2017). A mild dose of primary stimulus generates priming memory in plants that aids in overcoming harsh environments *via* metabolic changes (Singh et al., 2020). This work sheds light on how priming with *Trichoderma* induces defence priming in wheat against spot blotch and reduce the yield loss in wheat. *Trichoderma-*primed wheat showed enhanced vegetative growth as well as greater resistance under disease pressure as compared to non-primed wheat.

In the present study we evaluated the impact of *Trichoderma* priming on seed germination percentage of wheat. The results showed the reduced seed germination in primed wheat as compared to non-primed wheat (**Figure 1**). The reduced seed germination in primed seed may be due to the initial cost associated with priming. The reduction in percentage of seed germination is most likely due to the plant’s first perception of *Trichoderma* as stress, which may lead to the necessary modifications for priming for defence (Morán-Diez et al., 2021). Later on when the seeds were sown in the soil, the primed wheat showed enhanced growth as compared to non-primed wheat. All the results of growth parameters of primed and non-primed wheat suggest that *Trichoderma* priming enhances the vegetative growth in wheat. *Trichoderma* priming enhanced the shoot length, root length, total leaf area, shoot dry biomass and root dry biomass in wheat (**Figure 2**). *Trichoderma-*induced enhancement in aerial and radicular growth has been reported for a wide range of crop species (Singh et al., 2016; Aloisio et al., 2019; Moya et al., 2020). Such effects may be due to the capability of *Trichoderma* to increase nutrient availability, phosphate solubilisation and production of growth hormones such as Indole ascetic acid (IAA) in primed wheat (Singh et al., 2016). Primed plants showed fewer symptoms of spot blotch and decreased percent disease index as compared to non-primed, suggesting that primed plants were exhibiting better resistance to *B. sorokiniana* as compared to non-primed plants (**Figures 3A–F**).

In biochemical studies (**Figure 4**), level of photosynthetic pigments, ROS scavenging activity, total phenols and ascorbic acid was found to be almost similar in control (C) and only *Trichoderma* primed plants (T) indicating that there is no direct defense response by *Trichoderma* priming. Moreover, when these plants were challenged with *B. sorokiniana*, the primed one (T+B) performed better as compared to non-primed (B) suggesting that defence priming was activated only after challenge with *B. sorokiniana*. This confirms the establishment of defense priming rather than direct defense response due to *Trichoderma*. Under disease pressure primed plants (T+B) were performing almost similar to plants under no disease pressure (C, T) whereas non-primed plants (B) were found to be susceptible to *B. sorokiniana* upon challenge. Non-primed wheat showed a reduction in chlorophyll level after challenging with *B. sorokiniana* (**Figure 4A**). Such an effect may be due to degradation of chlorophyll molecules or may be due to inhibition of synthesis of new chlorophyll molecules (Roychoudhury et al., 2021). However, the primed plant showed an enhanced level of chlorophyll as compared to the non-primed under disease pressure. This may enhance the photosynthetic efficiency by increasing the channelization of light energy towards photosystems (Jisha and Puthur, 2016a). Similarly, Roychoudhury et al., 2021 also reported enhanced chlorophyll content in sodium nitroprusside primed seedlings of *Vigna radiata* under salinity stress. Carotenoid content was also found to be enhanced in primed wheat as compared to non-primed under biotic stress (**Figure 4B**). Carotenoid is an accessory pigment which may help in the protection of chlorophyll under stress conditions (de Cássia Alves et al., 2022).

Further, we evaluated the level of injuries in wheat caused by ROS under biotic stress by quantifying the MDA content. *B. sorokiniana* challenge increased the amount of MDA in non-primed wheat, while primed wheat had the same level of MDA as non-challenged plants suggesting that primed plants are almost as protected as non-challenged plants (**Figure 4C**). MDA is formed as a result of lipid peroxidation of the cell membrane, which leads to electrolyte leakage and ultimately to cell death (Basit et al., 2022). Reduction in MDA content has already been reported in BABA primed *Vigna radiata* and rice under abiotic stress (Jisha and Puthur, 2016a; Jisha and Puthur, 2016b). Seed priming with spermine also decreased the accumulation of MDA in rice under chromium stress (Basit et al., 2022). *Trichoderma* priming may have reduced the level of MDA by reducing the overproduction of ROS in wheat after pathogen challenge. Total ROS scavenging activity is the sum of all enzymatic and non-enzymatic antioxidant activities in plants under biotic or abiotic stress conditions (Sarker and Oba, 2018). Antioxidants balance the level of ROS inside cells by scavenging free radicals and improving plant defence responses (Espinoza et al., 2013). Primed wheat showed an augmented level of total ROS scavenging activity as compared to non-primed under disease pressure (**Figure 4D**), suggesting that *Trichoderma* priming enables wheat to maintain a high antioxidant level to prevent oxidative damaging of cells due to biotic stress.

Non-enzymatic antioxidants such as total phenols and ascorbic acid also play a role in plant protection against stresses (Sarker and Oba, 2018). In the present study, total phenol content was enhanced in both primed and non-primed wheat upon challenge with *B. sorokiniana* but primed wheat showed robust (1.57 times more) enhancement as compared to non-primed (**Figure 4E**). Similarly, the level of total phenol content was found to be enhanced in calcium compounds primed rice plants under fluoride stress (Singh et al., 2020). Augmented total phenol content may provide a better defence capability against *B. sorokiniana* in primed wheat. In the current investigation, the higher content of ascorbic acid detected in primed wheat as compared to non-primed under biotic stress suggests better performance of wheat under disease pressure due to *Trichoderma* priming (**Figure 4F**). An enhanced level of ascorbic acid was reported in potatoes showing tolerance towards *Phytophthora infestans* (Chung et al., 2019). In our study also, enhanced ascorbic acid may be responsible for tolerance in primed wheat against *B. sorokiniana*. In the present study all the biochemical changes are indicating that primed plants are performing better than non-primed after challenge with *B. sorokiniana*. This further added that priming could be beneficial strategy under hostile environment for crop improvement.

In the present study, *Trichoderma* priming has also improved the productivity of wheat measured in terms of the total no. of spikes, spike length, spikelet density, spikelet fertility, grain width, grain length, and grain weight after maturity. Yield parameters were found to be reduced in non-primed wheat challenged with *B. sorokiniana*. However, the primed wheat produced an almost similar level of yield to control with no disease pressure (**Figure 5**). Increased yield in primed wheat as compared to non-primed under disease conditions is most likely due to increased levels of photosynthetic pigment, total ROS scavenging activity, and non-enzymatic antioxidants (total phenol and ascorbic acid) upon pathogen challenge. Since there was no direct defence response due to priming stimulus, there was no wasteful consumption of metabolites for defence activation and hence no yield penalty in primed wheat. Das et al., 2021 have reported higher grain yield in rice by *Trichoderma* seed treatment under alternate wetting and drying irrigation. *Trichoderma* seed treatment in rice enables the plant to modify biochemical and physiological processes, allowing them to utilise resources (water, light, and nutrients) more efficiently and enhance yield performance (Doni et al., 2014). *Trichoderma* treated rice plants activate several photosynthetic enzymes, which increase photosynthetic efficiency and hence improve assimilate transfer from the shoot to the seed (Khadka and Uphoff, 2019). Translocation of more assimilates towards seed enhances grain yield in rice (Li et al., 2016). All the yield-related traits were enhanced in primed wheat as compared to non-primed suggesting that *Trichoderma* priming could be a sustainable tool for increasing the productivity of wheat.

The discovery of *Trichoderma*-mediated defence priming raises the important question of whether this response can be passed down to the next generation. Seeds were collected from all the four treatments of the G0 generation and sown in the pots. Interestingly, we found that Progeny (G1) of primed (T, T+B) wheat were showing better protection against *B. sorokiniana* in terms of reduced symptoms of spot blotch and percent disease index as compared to progeny of non-primed (C, B) wheat (**Figure 6**). The level of protection from spot blotch was almost similar in progeny of only *Trichoderma* primed wheat and progeny of *Trichoderma* primed and challenged wheat. Progeny of primed wheat, like their parents, demonstrated a heightened defensive response in terms of biochemical parameters (**Figure 7**). Enhanced levels of chlorophyll, carotenoid, total ROS scavenging activity, phenol, ascorbic acid and reduced levels of MDA were found in progeny of primed wheat as compared to progeny of non-primed wheat after *B. sorokiniana* challenge, indicating the establishment of IGIP. Yield performance was also found to be better in progeny of primed wheat as compared to progeny of non-primed wheat under disease pressure (**Figure 8**), suggesting that protection from defence priming is not just restricted to the plant’s lifetime, but also to its descendants. The biochemical and yield performance of primed wheat progeny plants were almost identical to that of non-challenged progeny plants, implying that progeny of primed parents are as protected as non-challenged plants. Rasmann et al. (2012) have demonstrated that herbivory makes Arabidopsis and tomato plants more resistant to successive attacks in the next generation through priming of JA-related defence responses. *Pseudomonas syringae* infection and b-aminobutyric acid treatment primed Arabidopsis for the activation of SA-mediated defensive responses in the next generation. (Slaughter et al., 2012). Another study discovered that the offspring of diseased Arabidopsis plants exhibited a faster and stronger SA-mediated defence response than controls (Luna et al., 2012). Ramírez-Carrasco et al., 2017 studied that transgenerational priming responses could last for at least two generations. Progeny of common bean primed with BABA exhibited increased resistance against *Pseudomonas syringae* pv. *Phaseolicola* by showing a high level of gene expression of the *PvPR1* gene (Ramírez-Carrasco et al., 2017). Spider mite herbivory also established a JA- dependent defence response for the next two generations in *Arabidopsis thaliana* (Singh et al., 2017). Caterpillar herbivory induced a defence response in wild radish within and across generations by inducing changes in the plant epigenome as well as physical and chemical defenses (Sobral et al., 2021). Overall, our present study clearly shows that *Trichoderma*-mediated priming induced inheritance is cost-effective and is a sustainable approach for wheat crop protection against *B. sorokiniana*.

## Conclusions

In order to cultivate enough food for a large population, we need to attain sustainable global food security in the current condition of rising chemical risks in nature. In our research, we discovered that pre-exposure of wheat to beneficial fungus, *Trichoderma*, initially incurs some associated cost in terms of reduced seed germination, but later on that cost was nullified and enhanced vegetative growth (root, shoot length, leaf area, and plant biomass) of the plants were recorded. Upon challenge with *B. sorokiniana*, *Trichoderma* primed wheat exhibited more resistance by showing enhanced levels of photosynthetic pigment, ROS scavenging activity and antioxidants as compared to non-primed wheat. Reduced levels of MDA indicated less cell damage in primed wheat as compared to non-primed, which consequently improved the productivity. Biochemical studies revealed that *Trichoderma* has induced priming in wheat but not mediated direct defence response. Like their parents, the progeny of the primed wheat also showed enhanced resistance against *B. sorokiniana* as well as higher productivity under disease conditions as compared to non-primed wheat indicating that priming is not confined within generation but also passed on to the next generation. Given that priming provides long-lasting, stress resistance, plant defence priming could be a non-pesticide alternative with broad effectiveness. It could play a promising role in integrated pest/pathogen management and provide a sustainable approach to crop protection. An efficient induction of IGIP would allow poor farmers to collect their own seed stocks of more resistant crop varieties, thereby making their food production less vulnerable to outbreaks of pests and disease. As a result, *Trichoderma-*mediated defence priming is therefore of great importance in the sustainable agricultural practices and crop production.

## Data availability statement

The original contributions presented in the study are included in the article/**Supplementary Material**. Further inquiries can be directed to the corresponding author.

## Author contributions

PS and MT conceived the idea. MT, RS performed the experiment. MT, PS prepared the manuscript. MT, RJ and PS edited and finalised the manuscript. All authors contributed to the article and approved the submitted version.
