# Supplementary material for: Heritable priming by Trichoderma: A sustainable approach for wheat protection against Bipolaris sorokiniana
Source: Front Plant Sci. 2022 Dec 16;13:1050765. doi: 10.3389/fpls.2022.1050765 (PMC9807111; doi:10.3389/fpls.2022.1050765)
Supplement: Supplementary file 1 [file DataSheet_1.pdf]

Supplementary figures

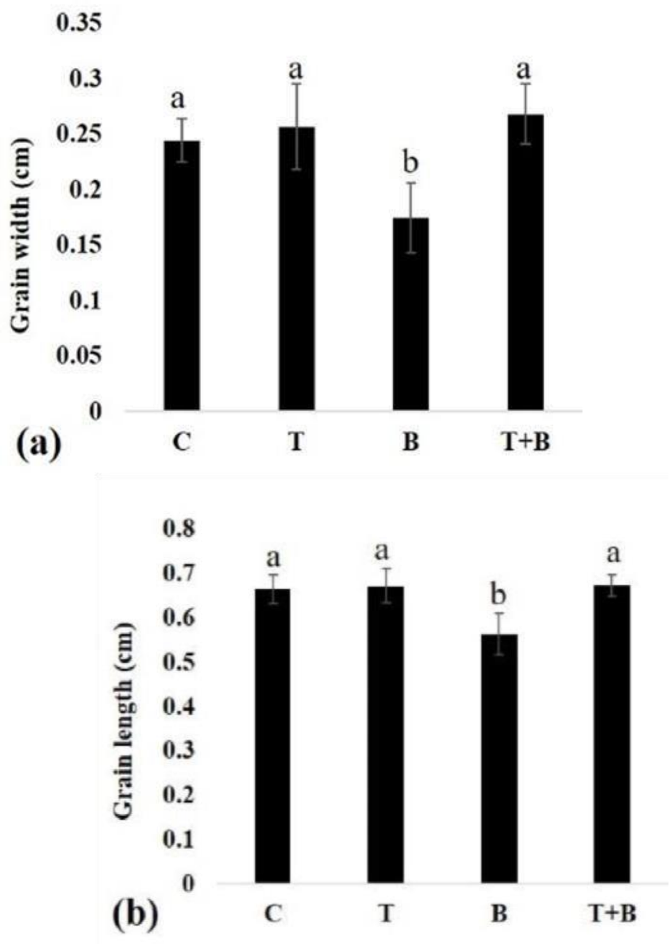

Figure 1- Effect of *Trichoderma*-priming on the yield of the wheat in G0 after challenging with *Bipolaris sorokiniana* HD 3069. **(a)** Grain width. n = 25, **(b)** Grain length. n = 25.

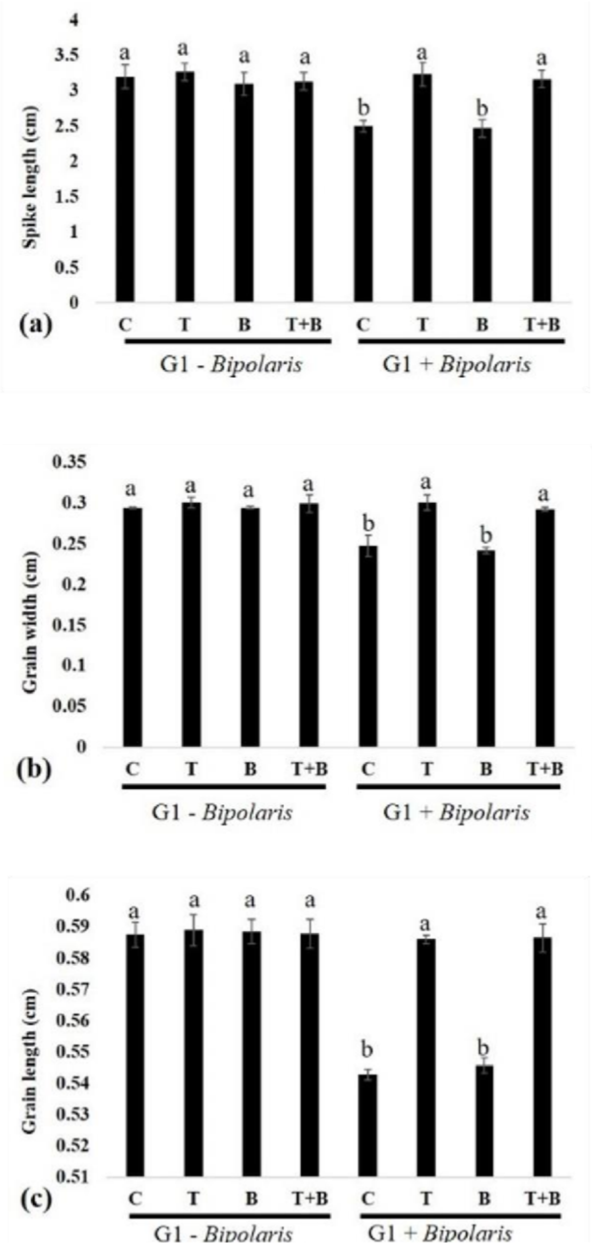

Figure 2- Effect of IGIP on the yield of the wheat in G1 after challenging with *Bipolaris sorokiniana* HD 3069. **(a)** Spike length, n =20, **(b)** Grain width. n = 25, **(c)** Grain length. n = 25.
