# Supplementary figures and images for: Heritable priming by Trichoderma: A sustainable approach for wheat protection against Bipolaris sorokiniana
Source: Front Plant Sci. 2022 Dec 16;13:1050765. doi: 10.3389/fpls.2022.1050765 (PMC9807111; doi:10.3389/fpls.2022.1050765)

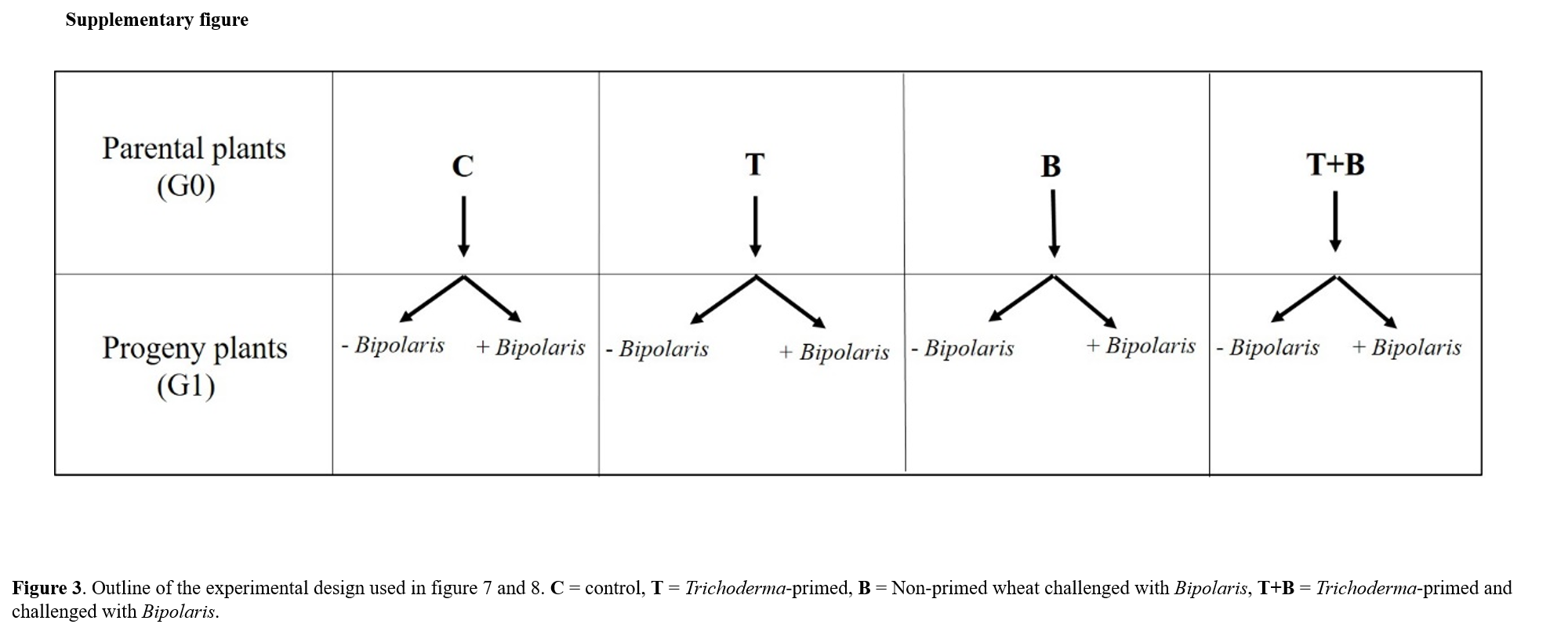

Supplement: Supplementary file 2 [file Image_1.tif]
